# The topology of representational geometry

**DOI:** 10.3389/fnins.2025.1597899

**Published:** 2025-06-20

**Authors:** Shael Brown, Reza Farivar

**Affiliations:** ^1^Department of Quantitative Life Sciences, Farivar Lab, McGill University, Montreal, QC, Canada; ^2^Department of Ophthalmology and Visual Sciences, McGill Vision Research, McGill University, Montreal, QC, Canada

**Keywords:** representational similarity analysis, topological data analysis, persistent homology, representational geometry, object representation, human, macaque, fMRI

## Abstract

Representational similarity analysis (RSA) is a powerful tool for abstracting and then comparing neural representations across brains, regions, models and modalities. However, typical RSA analyses compares pairs of representational dissimilarities to judge similarity of two neural systems, and we argue that such methods cannot capture the shape of representational spaces. By leveraging tools from computational topology which can probe the shape of high-dimensional data, we augment RSA to be able to detect more subtle yet real differences and similarities of representational structures. This new method could be used in conjunction with regular RSA in order to make distinct, complementary inferences about neural function.

## 1 Introduction

Comparisons of representations in human cortex across modalities, brain regions, or between models and brain responses, can give meaningful insight into the neural mechanisms which encode them, and *representational similarity analysis* (RSA) is a popular framework for organizing and analyzing many such comparisons. RSA estimates the *representational geometry* of a (neural) computational system as a matrix of representational similarities (RSM) or dissimilarities (RDM) (Kriegeskorte and Diedrichsen, 2019). Two such matrices can then be compared with a *second order isomorphism* to quantify similarity or differences between the two systems—Spearman correlation is commonly used in neuroimaging studies (Shepard and Chipman, 1970; Kriegeskorte et al., 2008a). Correlation between RDMs can identify (i) brain regions which similarly represent stimuli, (ii) commonalities in neural codes between species, (iii) computational models which faithfully represent the function of a brain region, and more.

The powerful and flexible machinery of RSA has yielded many successes in neurosciences and neuroimaging in particular—the introductory paper (Kriegeskorte et al., 2008a) has been cited over 3,300 times so far. More pertinent to the current discussion are examples of RSA applied to fMRI vision studies—one study (Connolly et al., 2012) showed that there may be a spectrum of representations of animals in human visual cortex, from most animate to least; another study (Bracci and Op de Beeck, 2016) showed that visual areas represent shape and category to different extents and with interactions; and in Kriegeskorte et al. (2008b) it was shown that primates may have a similar neural code for object representations in the IT cortex.

**Figure 1:**
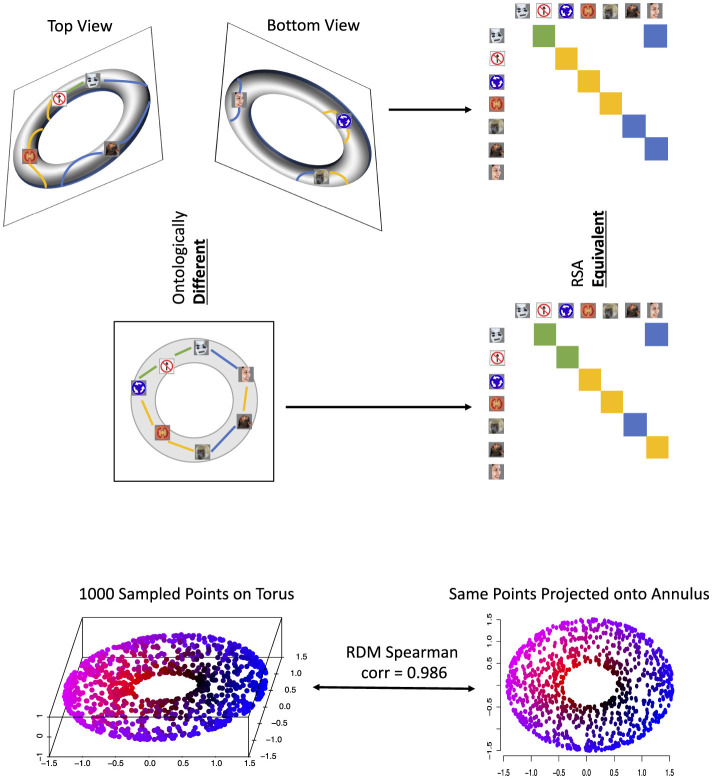
**Top:** seven sampled representations from a torus (3-D object with two loops), projected onto an annulus (2-D object with only one loop), and the resulting two RDMs. The stimuli images were obtained from the supplemental information in (Kriegeskorte, 2009) but were originally introduced in (Kiani et al., 2007), as is the case in later figures. **Top left** are the top and bottom views of seven sampled representations from the surface of the torus, with colored lines indicating representational distances between adjacent points (green for small distances, yellow for medium and blue for large). **Middle left** is the projection of these torus representations onto an annulus, with updated representational distances. These distances for both shapes are color-coded in their respective RDMs, which would be considered equivalent by RSA, despite the representational spaces having completely different shapes. Bottom: a simulation of 1,000 points sampled from the torus (3-D object; **bottom left**), projected onto an annulus (2-D object; **bottom right**) and the Spearman correlation of their resulting RDMs—0.986, again showing the erroneous equation of two totally different topologies.

However, there have been several major criticisms of RSA, the strongest being that two computational systems with highly similar RDMs may be carrying out their computations in fundamentally different ways (Dujmović et al., 2022; Chen et al., 2021). With the primary goal of RSA being to infer whether two computational processes are similar or not, this issue alone may limit our inferences from RSA experiments. For example, at the top of Figure 1 we consider a representational space of a torus, i.e., a hollow doughnut, with seven sample representations. Projected onto two dimensions (for instance using multidimensional scaling) the torus becomes a 2D annulus (i.e., a circle with added noise) and representations sampled from the torus project to representations in the annulus. Yet the RDMs of the two sets of representations, one set on the surface of the torus (in 3D) and the other along the 2D annulus are erroneously equated by RSA. The same logic also holds with a larger number of sampled representations - as seen in a simulation of 1,000 points sampled from a torus at the bottom of Figure 1 where the Spearman correlation of the two 1000-by-1000 RDMs was 0.986. This example demonstrates that the central assumption of RSA—that linear distances of matrices sufficiently captures similarity of representational geometry for comparison—is not necessarily true.

While our example may seem artificial, the population of orientation-selective neurons in V1 together have the representational space of a loop (Singh et al., 2008), while rat grid cells have the representational space of a torus (Curto, 2017). Thus we must use tools that are sensitive to the shape of the representational space of neurophysiological data, or we may err in drawing similarities between two cells (e.g. a group of grid cells and a group of V1 simple cells) based on a simplified model of their representation.

But does this example demonstrate a problem using RDMs to capture representational geometry or rather a problem using correlation as a second-order isomorphism? It has previously been suggested that using non-linear second-order isomorphisms would better account for non-linear geometries (Kriegeskorte and Kievit, 2013), and some studies proposed such isomorphisms for analyzing correlation matrices of neurological data (Shahbazi et al., 2021; You and Park, 2022). A technique called distance correlation (Szekely et al., 2008) has also been shown to be a useful measure of independence in RSA model comparisons (Diedrichsen et al., 2020), being able to capture non-linear dependencies as well as linear ones.

These approaches account for the non-independence of pairs of correlation/distance matrix entries, but in the example offered above comparing a torus and its projection onto an annulus, the difference comes from global topology, which implicates distinct causal mechanisms—the annulus contains one periodic phenomenon (captured in one loop) whereas the torus contains two—A torus can be described by two loops: the major loop, which goes around the central hole, and the minor loop, which circles around the tube itself. Such structural features are only detectable when taking into account all dissimilarities together, not just the non-independence of pairs. For example, a Gaussian-distributed cluster, and the same cluster punctured with a small hole in its center, will have similar covariance matrices despite the former being a cluster and the latter being an annulus.

Unfortunately it is not possible in RSA to segment RDMs into features (i.e., sub-components) of their representational geometries. Multidimensional scaling (MDS) (Mead, 1992) has been used to project RDMs into lower dimensions for visualization of representational spaces (Kriegeskorte et al., 2008a), but the projection dimensions are not directly interpretable and are always linear. For example, in (Kriegeskorte et al., 2008b) RSA found evidence of a shared neural code in primate IT cortex, but MDS embeddings only revealed a blob-like distribution of representations coarsely separated by object category. If segmentation of representational spaces were possible, we could have linked representational similarity to features of the representational spaces (see below), but this is not possible with current RSA methods. In summary, RDMs, and RSA by proxy, do not address complex (i.e, global and not linear) representational geometries and the question of appropriate second-order isomorphism may only be solved once the representational geometry is appropriately captured.

The mathematical discipline concerned with studying distance/adjacency between objects in an abstract space is *topology* (Hatcher, 2002), and tools from computational topology can be applied to multivariate data to derive topological metrics. Structure or shape of data at a local scale can be integrated into global shape descriptors using topological tools, and shape features can then be quantified and analyzed to capture the richness of data structure. Topology has a number of desirable qualities for analyzing representational geometries. For example, the topology of an object does not change when the object is rotated, stretched or reflected (Hatcher, 2002). Robustness to small amounts of these transformations in data would also be expected of representational geometries—the features of neural codes do not depend on the order of the labels of functional units, or the scale of neural activity (Laakso, 2000).

The field of *topological data analysis* (TDA) provides practical tools for analyzing data using topology, and TDA has been applied in myriad fields (Carlsson and Vejdemo-Johansson, 2021). The most established tool in TDA is *persistent homology*, PH (Edelsbrunner et al., 2000; Zomorodian and Carlsson, 2005), which seeks to identify topological features present in data, classified by their dimension. Persistent homology takes as input the distance matrix of a dataset (like an RDM), and identifies structures inherent in the data, such as clusters (i.e. disconnected components), loops and voids (which represent elements of the *H*_0_, *H*_1_ and *H*_2_ groups respectively). Persistent homology also provides information about the the sizes and density of points belonging to these structures, which can be used to determine which features are significant.

**Figure 2:**
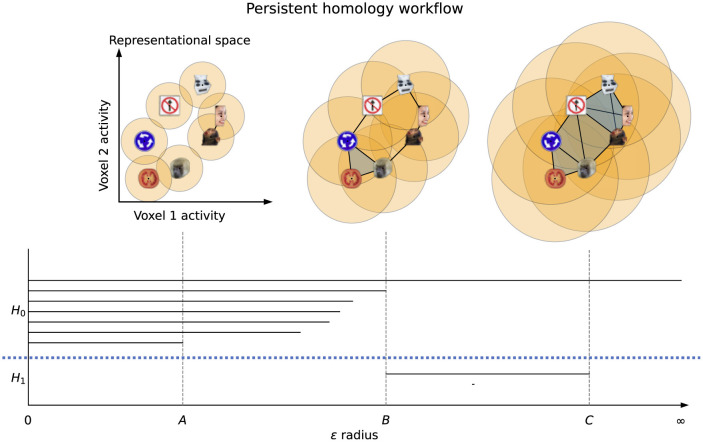
Persistent homology workflow. A linkage radius ϵ is increased from 0 and representations (i.e., data points) are connected when their distance is at most ϵ, forming Vietoris-Rips complexes. Seven clusters and two loops are present in the dataset, and are tracked by the PH algorithm with each having its own line segment. *H*_0_ and *H*_1_ are separated by the dotted blue line. At linkage radius *A* there are six clusters (since the human face and monkey face are connected, and hence one cluster has died off), while at radius *B* the loop is fully connected (and all components merge into one) and at *C* the loop is filled in (i.e., is no longer a loop).

**Figure 3:**
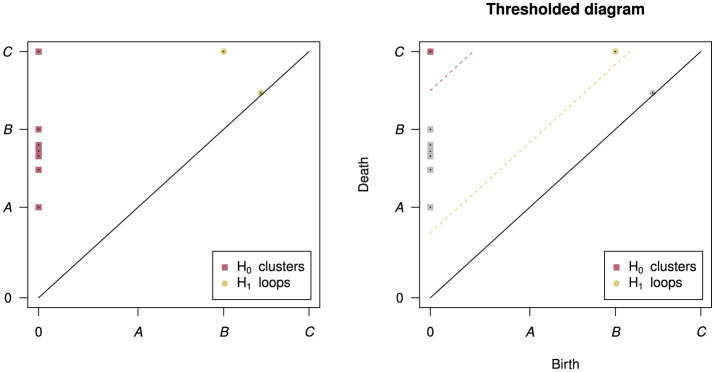
The output persistence diagram of PH run on the example dataset in Figure 2 **(left)** and an example thresholded diagram **(right)**. In the persistence diagram there are points for each of the seven clusters and two loops - one loop is very close to the diagonal line where birth and death are the same, indicating that this loop was very “short-lived”. In the thresholded diagram only one cluster and one loop were significant, indicated by their color and placement above their respective threshold lines.

The workflow of the PH algorithm can be seen in Figure 2. The process begins with a sweep through values of a linkage radius—a parameter that defines the extent of the neighborhood within which two data points would be joined (linked) to form a structure (called the *Vietoris-Rips complex*), and the topological features of these structures at each linkage value are classified as belonging to either *H*_0_ (clusters), *H*_1_ (loops), *H*_2_ (voids), etc. As we sweep through linkage values, features will appear, persist across some range of linkage values, and then disappear—as shown in Figure 2, the points on the loop form that loop only within a certain range of radii *B* and all points eventually fully connect at radius *C*, destroying the loop structure. The linkage values where a feature comes into existence and ceases to exist are called the birth and death values, respectively. For further mathematical details on this process see (Edelsbrunner et al., 2000; Zomorodian and Carlsson, 2005). The birth and death values, along with the feature dimensions, are plotted in persistence diagrams as shown in Figure 3, and points that have death value much larger than their birth value (i.e. their point in the persistence diagram is high above the diagonal line where birth and death are equal) are called "persistent"–they live long and prosper. A thresholding procedure (Fasy et al., 2014) can then be used to distinguish between persistent (i.e. significant) topological features and non-persistent (i.e. noise) topological features, and an example of this can also be seen in Figure 3. While less persistent features in a persistence diagram may capture some data signal (Bubenik et al., 2020) more persistent features are robust to subsampling, and are therefore more stable to analyze. Another useful piece of information that can be extracted for each topological feature is the *representative cycle*, which is a subset of data points in a given topological object. For additional details, see (Chazal and Michel, 2017).

As the number of sampled points in an object grows, the topological features of the points, recovered by persistent homology, converge to the underlying features (Chazal et al., 2014) even in the presence of noise in the dataset (Edelsbrunner et al., 2000). In this sense, increased sampling provides greater validity of the topology of the data space.

It has been demonstrated that persistent homology can detect event-related periodic spatial signals (i.e. spatial loops) in simulated event-related fMRI data (Ellis et al., 2019). A related technique for calculating persistence diagrams called “persistent cohomology” can detect representational space topologies of neural population responses of simulated rat neurons (Kang et al., 2021). Persistent homology has also been used to find meaningful structure in correlation matrices of spike trains in rat place cells using vectorized summaries of persistence diagrams called Betti curves (Giusti et al., 2015). PH also correctly characterized a low-dimensional neural manifold of mouse behavior analyzing binned spike counts of thalamic neurons (Chaudhuri et al., 2019). The results of these studies suggest that persistence diagrams are a useful tool for characterizing representational spaces, but four properties of diagrams make them particularly well-suited for this task:

1. The topological features in persistence diagrams can be identified in their input datasets, up to a choice in representative cycle, thereby allowing us to segment datasets into representational features.
2. Persistence diagrams remain consistent under different orderings of the same variables.
3. Two persistence diagrams can be meaningfully compared even if their input datasets contained different numbers of data points or variables.
4. Persistence diagrams converge as the number of data points in their input datasets grow Chazal et al. (2014).

Property 1 means that we can uncover topological features of representational geometries, allowing for constraints on the mechanisms implicated while making comparisons between systems more interpretable. For example, a torus and a loop have different numbers of significant loops (two and one respectively), and therefore we could distinguish between RDMs sampled from them. Also, two systems with similar linear aspects in their geometries may perform different calculations and this could be uncovered by investigating their topological sub-structures.

On the other hand, Properties 2 and 3 suggest that using persistent homology to analyze representational geometries may allow for the pooling of data from different studies, even studies with different (but relatable) sets of conditions/stimuli so long as their pooling is defensible and interpretable to the researcher—for examples, studies investigating face processing may use different face conditions, different non-face stimuli, etc, and Properties 2 and 3 of PH allow us to pool results from these studies for stronger inference of representations. It should be noted, however, that quantitative comparisons of persistence diagrams, such as distance metric calculations, will be confounded to some degree by the sizes of the input diagrams, so future work should establish guidelines for comparing different datasets sizes with RTA.

As multiple RDMs can be compared using RSA, we would need an equivalent topological tool to compare multiple persistence diagrams. For second-order isomorphisms of persistence diagrams there exist two main approaches in the literature—for differences, we can use distance calculations (Kerber et al., 2017) and for similarities we can use kernel calculations (Le and Yamada, 2018). Since topological features can be comprised of any number of data points (representations), we can capture differences between any number of data points between two representational geometries using these topological second-order isomorphisms. While in regular RSA differences and similarities are essentially opposites (like in the case of correlation and correlation distance), due to the complex nature of persistence diagrams (Turner et al., 2014) we need specialized and distinct tools to calculate their differences and similarities.

Two typical analyses of RDMs include

- Inference—deciding if two RDMs or two groups of RDMs are similar/different (an important example of which is model comparison), and
- Visualization—using MDS to project an RDM into low dimensions (Kriegeskorte et al., 2008a).

Similar analyses can be performed with persistence diagrams—differences among sets of persistence diagrams can be found using distance-based permutation approaches as in Robinson and Turner (2017); Abdallah et al. (2023)—and the pairwise distances between multiple persistence diagrams can be used to form an MDS embedding of the diagrams into a low-dimensional space. We have implemented these analytical and inferential tools to carry out TDA on large multivariate datasets (e.g. fMRI) in our software package TDApplied Brown and Farivar-Mohseni (2024). Therefore, the machinery is in place to analyze persistence diagrams computed from RDMs in ways similar to RSA.

We propose a new approach called *representational topology analysis* (RTA) for detecting structures of representational space. In RTA, RDMs are converted to distance matrices (although this is not necessary for correlation dissimilarity matrices; see the methods section) and then analyzed with persistent homology, resulting in *persistence diagrams*, i.e. *representational topologies*, that can then be analyzed with topological machine learning and inference methods. Comparing persistence diagrams is preferable to comparing RDMs because the latter do not encode the topology of data space, while the former explicitly represents this information. Representational topology analysis is ideal in conjunction with regular RSA (for inference on linear aspects of data space) in order to make powerful inferences about representational geometry and, by extension, fundamental mechanisms that gave rise to them. Interpretations from RTA are also complementary to interpretations from RSA because in the topological case we can make conclusions about when two representational geometries are different or similar topologically, compared to the regular RSA case where we can only say if two geometries are linearly different or similar. Below, we applied RTA on two datasets and were able to answer questions that regular RSA could not, demonstrating the potential value of representational topology.

A related framework to RTA in the literature is topological RSA (tRSA) (Lin and Kriegeskorte, 2024), which aimed to (i) permit a trade-off in studying representational geometry and topology, and (ii) abstract from the noise and individual idiosyncrasies of representational geometries using topology. In tRSA the values in RDMs were transformed according to special monotonic transformations, resulting in new data structures called representational geo-topological matrices (RGTMs), which were then compared with Euclidean distance. By comparing tRSA and RSA on several simulated topological datasets, the authors found that tRSA was better able to resolve representational topology compared to RSA. On the other hand, in a visual fMRI study it was found that tRSA was no better at finding regionally consistent (topological) signatures of computation across subjects than RSA. While tRSA shows promising flexibility in studying representational topology and geometry simultaneously, unlike RTA it does not compute a true topological descriptor of the dataset (like the persistence diagram) and therefore cannot provide insights into the topological, previously unseen features of neural computation that RTA can provide. Also, we will show several scenarios where RTA does outperform RSA on certain tasks.

One of the most important features of RSA is the ability to carry out model comparison (i.e. adjudication) (Kriegeskorte et al., 2008a). A correlation between two RDMs admits a p-value, capturing the significance of the linear relationship between them. Therefore, given a list of candidate “model” RDMs for a “target” RDM, the candidate with highest statistically significant correlation with the target would be considered the “best model”.

**Figure 4:**
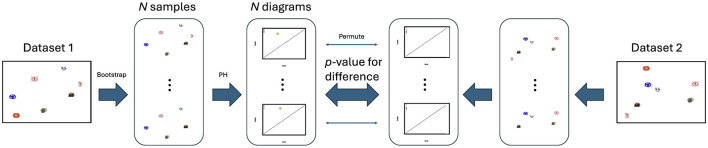
A new model adjudication procedure for RTA. The stimulus set are repeatedly bootstrap resampled *N* times, and for each resampling we subset both RDMs and compute the two bootstrap persistence diagrams. This process results in two groups of persistence diagrams, one for each representational dataset, with a correspondence across the groups established by the shared stimulus subsamples. We then use a permutation test to determine if there is a statistical difference between the two datasets based on a measure of within-group variation, and this test procedure returns a *p*-value. A lower p-value indicates less similar representational spaces, and a higher *p*-value indicates more similar spaces.

Here we propose a novel method for statistical inference on a target dataset *T* and a list of candidate model datasets {*M*_*i*_}, in terms of topological similarity. This method integrates two established procedures from the applied topology literature—a bootstrap procedure (Fasy et al., 2014) and a hypothesis testing procedure for group differences (Robinson and Turner, 2017). The concept for our idea is displayed graphically in Figure 4.

If two representational spaces are similarly and sufficiently sampled from the same shape structure then the resulting two sub-representational spaces should have similar shape features. We therefore estimate the sampling distribution of the persistence diagrams for each pair of the two spaces (*T, M*_*i*_) by repeated bootstrap resampling (i.e. sampling with replacement)—each resample results in a persistence diagram for that dataset (i.e. *PD*_*T*_*sub*__ and *PD*_*M*_*i, sub*__). We then quantify the pairwise topological distance between each of these resample pairs using the bottleneck distance (Kerber et al., 2017). The sum of the upper triangle represents the topological analog of the variance estimates in classic univariate statistics, and the sum of these distances for the two datasets is analogous to the mean sum of errors. To determine if *T* and *M*_*i*_ represent statistically distinct topologies, we use a permutation test whereby we determine the probability of obtaining a smaller sum of distances from chance—we shuffle each pair, *PD*_*T*_*sub*__ and *PD*_*M*_*i, sub*__, between the two groups (Abdallah et al., 2023) and calculate the sum of distances for the sample, *N* times. The frequency of permuted distances being less than our test statistic (the sum of distances of the subsampled diagrams) determines the chance probability of obtaining such a difference. This gives a p-value of topological difference in each desired topological dimension between each pair of spaces (*T, M*_*i*_).

For a given p-value threshold we can determine which comparisons pass and do not pass this difference test, and out of all comparisons that are not significantly different we wish to know which *M*_*j*_ is the “best model” of *T*. We calculate a 95% confidence interval for the topological distance between *T* and *M*_*j*_ by computing the distance between each *PD*_*T*_*sub*__ and *PD*_*M*_*j, sub*__ from each bootstrap subset in the previous step. The 2.5% and 97.5% percentiles of these values give the bounds of the 95% confidence intervals. Any *M*_*j*_ with a confidence interval fully below that of another *M*_*k*_ is a better model of *T*.

The two steps we outlined—permutation testing followed by confidence interval generation—is our model inference procedure.

The aforementioned model adjudication procedure allows us to make comparisons between representational topologies, but we still need a tool to help us compare a representational topology with a representational geometry (i.e. to compare RTA with RSA). To this end we introduce a novel visualization technique, called the proximity labeled rips graph (PLRG), which displays a snapshot of the topological structure of a representational dataset and uses colors to show which topological features were captured by geometry.

Our PLRG builds on the *Vietoris-Rips graph* (Zomorodian, 2010) or VR graph for short. At each linkage radius ϵ in persistent homology, a set of edges are defined between the data points based on their distances (all the distances ≤ ϵ), and this defines a graph at each linkage. In our analyses we will generally compute VR graphs at the birth scale of the most persistent loop. To get a PLRG from a VR graph we color the graph nodes by the MDS embedding coordinates of each corresponding point in the RDM—similar colors indicate geometric proximity, and therefore color gradients in part of a PLRG suggest an agreement between RSA and RTA in that subset of data, and vice versa.

There are, of course, other popular techniques for visualizing high-dimensional data as a graph, including Isomap (Tenenbaum et al., 2000), UMAP (McInnes et al., 2018) and the Mapper algorithm (Singh et al., 2007; Saggar et al., 2022, 2018). Isomap is the most similar to our approach, with only a different graph layout algorithm—an MDS projection of the path distance matrix between each pair of nodes in the neighborhood graph. Therefore, substituting the VR graph for an Isomap graph would only potentially produce qualitatively different plots, which we did not feel was important to test for comparison. UMAP, on the other hand, constructs a quantitatively different neighborhood graph based on asymmetric *k*-nearest neighbor calculations—a graph which does not capture the topological connectivity at a single ϵ radius from persistent homology. Finally, Mapper graphs mix the node coloring (which represents representational *geometry* in the PLRG) and the graph construction procedure (which represents representational *topology* in the PLRG) via filter functions, whereas the PLRG keeps geometry and topology distinct for comparison. For these reasons we do not compare our PLRGs against UMAP or Mapper graphs.

## 2 Materials and methods

In order to compare RTA with RSA we carried out two studies—the first used data from one of the seminal studies of RSA (Kriegeskorte et al., 2008b), and the second used data from a study of shared representations of naturalistic movie viewing across subjects (Zhang and Farivar, 2020; Hall, 2009).

### 2.1 Model adjudication

In both of our analyses we compared pairs of representational spaces to look for topological differences. We used the “permutation_inference” function from TDApplied with 100 bootstrap samples and 1,000 permutation iterations. Since, for each representational comparison, there was exact correspondence between rows (and columns) of the two RDMs we used the paired inference procedure for distance matrices, and the maximum radius we used to calculate the bootstrapped persistence diagrams was the joint maximum representational dissimilarity contained in the two RDMs.

### 2.2 Human, monkey, and GoogLeNet comparison

**Figure 5:**
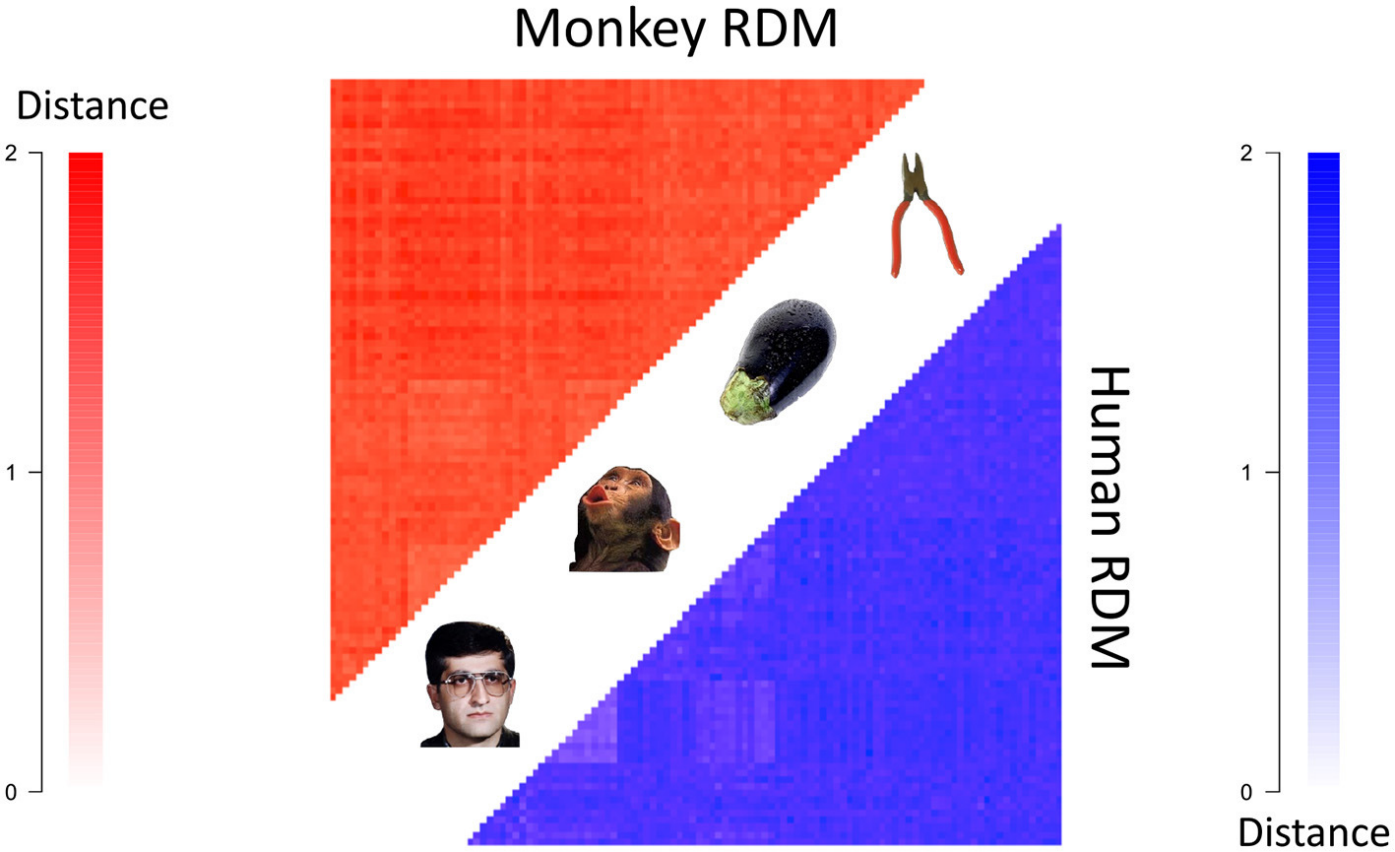
The mean human **(bottom right)** and monkey **(top left)** RDMs (each converted to a distance matrix using the transformation $1-\rho \to \sqrt{2*\left(1-\rho \right)}$). Darker colors indicate greater representational distances.

One of the earliest applications of RSA to visual fMRI studies was (Kriegeskorte et al., 2008b), in which RSA was used to show a common representational code in the primate inferior temporal cortex by comparing fMRI data in humans and electrophysiology data in monkeys [which was collected in the study (Kiani et al., 2007)]. But what topological representational features exist in this shared space? Regular RSA cannot segment RDMs to find features of a representational space, and therefore cannot address this question. One of the authors of (Kriegeskorte et al., 2008b) provided us with the mean RDMs from the group of four humans and the group of two monkeys for the 92 visual stimuli displayed in Figure 5. The stimuli in the experiment were images of various categories, including animals, humans, body parts, naturalistic scenes and objects, and these images can be found in the supplementary data of Kriegeskorte et al. (2008b).

We received two RDMs from Kriegeskorte et al. (2008b), one which was the average RDM from four human subject's 3T fMRI data and the other of which was the average RDM from two monkey subject's electrode recording data. The entries of the RDMs were (average) correlation distances (i.e. 1 subtract Pearson correlation) between the spatial response patterns of voxels/cells for each pair of stimuli. For more details, see Kriegeskorte et al. (2008b). We further transformed the correlation distance values from 1−ρ to $\sqrt{2\left(1-\rho \right)}$ which better satisfy the mathematical notion of distance Brown and Farivar-Mohseni (2024).

We calculated persistent homology of the two RDMs using the R package TDA (Fasy et al., 2021), up to homological dimension 1 (loops), up to the connectivity radius which was the maximum RDM entry, and using the dionysus library functionality (Morozov, 2017) to calculate representative cycles (i.e., a subset of the data points that lie on each loop) for the loops. We then computed VR graphs (Zomorodian, 2010) from the two RDMs at the scale of the birth radius of the most persistent loop for each RDM. The stimuli in the two representative cycles were highlighted with deeper colors. The layout of the graph is optimized to project connected nodes nearby each other in 2D space and unconnected nodes further apart, using the default Fruchterman-Reingold (Fruchterman and Reingold, 1991) graph layout algorithm from the R package igraph (Csardi and Nepusz, 2006). We plotted only the graph component which contained the representative cycle nodes because there was a significant jump in the persistence value (roughly 24% in the human diagram and roughly 15% in the monkey diagram) from the most persistent loop to the next most persistent loops (the next number of jumps in both diagrams were a difference of less than 1%). In cases where there are multiple long-lived loops of similar persistence values it is important to note that their exact ordering may be due to noise in the input dataset, and the most persistent loop (and its representative cycle) may not be stable. The computation and visualization of the VR graphs was performed by TDApplied.

The six GoogLeNet RDMs were computed from the layer activations of the GoogLeNet model (Szegedy et al., 2015) applied to the same set of image stimuli as in Kriegeskorte et al. (2008b). The RDMs also contained transformed correlation distances of activations, like the human and monkey RDMs. The same plotting procedure was used to produce the GoogLeNet PLRGs, and in the four layers with at least two loops—the fourth, fifth and final layers (the first layer had one loop)—the jump in persistence value from the most persistent loop to the next most persistent loop was 15%, 103% and 9% respectively.

All combinations of the human, monkey and GoogLeNet RDMs were compared with our model inference procedure.

### 2.3 Naturalistic movie viewing study

In Zhang and Farivar (2020), local spatial patterns of BOLD activity in subjects viewing 2D and 3D naturalistic movies (Hall, 2009) were found to be highly conserved across subjects in early visual areas and were modified by region and visual stream—early, ventral and dorsal. It would therefore be expected that group-average topological features differ by region especially for regions in different visual streams. In order to test this hypothesis we analyzed region-level data from Zhang and Farivar (2020), constructing timepoint-by-timepoint spatial-pattern correlation distance RDMs (i.e., the correlation distance between time *i* and *j* of the BOLD patterns in each region) and used RTA to characterize the shape of representational space in group average RDMs from certain early, ventral and dorsal regions.

For a detailed account of the data, acquisition and preprocessing of our naturalistic movie viewing analysis, see Zhang and Farivar (2020). The study collected 3T fMRI data, with 3mm^3^ voxels, from 55 subjects watching four 5-min movie clips in one scan (two clips each viewed in both 2D and 3D). The TR was 2 s, and the first 1 minute of each movie clip was not analyzed, resulting in 120 TRs of data for each movie clip. Data preprocessing was carried out with the AFNI software (Cox, 1996) and fMRI voxel data was projected onto cortical surface nodes (36,002 per hemisphere) with the SUMA (Saad and Reynolds, 2012) and FreeSurfer (Fischl et al., 2002) software packages. Cortical regional boundaries followed the probabilistic atlas from Wang et al. (2015).

Movie viewing is a naturalistic task that typically induces very similar temporal patterns of activity in a group of subjects Hasson et al. (2004, 2010) and it has recently been shown that this similarity is likely driven by gamma oscillations (Chen and Farivar, 2020) and is detectable in the spatial patterns in a manner that is more informative of viewing condition (stereoscopic 3D vs mono) than the temporal pattern correlation (Hasson et al., 2004). Here, we used RTA to determine whether the structure of the representational space of spatial patterns over time is different between regions/streams.

To this end we computed group-average region-level significant topological features—loops which survived the thresholding procedure of Fasy et al. (2014) (see the methods section for details). Significant loops which exist at different scales (i.e. with different birth and death values) would indicate qualitatively different representational structures. We computed the mean RDMs for five regions, across subjects, of higher ventral regions VO1 and VO2, higher dorsal regions PHC1 and PHC2 and the early region V3 in both hemispheres for both 3D movie clips, resulting in 20 RDMs. We chose to analyze only 3D movie data to ensure that there was no confounding effect of stimulus condition in our analysis, and because 3D movies are closer to naturalistic stimuli than 2D movies. We used the bootstrap procedure to identify statistically significant loops, and determined the most persistent significant loops from the VO regions, PHC regions and V3. The result was three group-average RDMs—one VO RDM, one PHC RDM and one V3 RDM.

We chose to solely analyze 3D movie clips in our analysis, in the regions V3, VO and PHC. To calculate an ROI RDM in a hemisphere for a particular movie clip we selected the surface nodes in that hemisphere which were in the ROI (based on atlas boundaries), and computed Pearson correlation between each pair of TRs of the time series activity of all the nodes in that movie. This resulted in a 120x120 representational similarity matrix, which was converted to an RDM by transforming each correlation value ρ to the distance value $\sqrt{2\left(1-\rho \right)}$. To obtain a group average RDM for each region, movie and hemisphere, we averaged the subject-specific RDMs.

We calculated persistent homology of the RDMs using the R package TDAstats (Wadhwa et al., 2019), up to homological dimension 1 (loops) and up to the connectivity radius which was the maximum RDM entry. This homology calculation was used in conjunction with the bootstrap procedure (Fasy et al., 2014) in TDApplied to identify significant topological features, and was implemented with 30 bootstrap iterations and significance threshold α = 0.1 to avoid over thresholding. We chose to use thirty samples because it is the generally accepted minimum guideline for statistical inference methods like the *t*-test (Kwak and Kim, 2017). The full persistence diagrams for V3, VO and PHC contained, on average, roughly 276 loops, compared to 0.1 loops on average in subsetted diagrams after thresholding. The subsetted persistence diagram, according to the bootstrap thresholding procedure, then contained significant group-average region-level topological features (loops). For each region—V3, VO and PHC—we identified the most persistent significant loop out of all its thresholded diagrams, the loop's birth radius and the RDM it came from. We then used the R package TDA to calculate the representative cycles for those three significant loops from their respective RDMs, by performing the same persistent homology calculation with the dionysus library functionality. We then used our model adjudication procedure to compare each of the three regional topological spaces.

Our novel Proximity-Labeled Rips Graph (PLRG) visualization requires an RDM, the birth scale of a loop and its representative cycle. The nodes of the PLRG graph are the TRs (i.e. spatial patterns) and connections between nodes are determined by the RDM entries which are at most the birth scale (i.e. a PLRG is a VR graph). We plotted only the graph component that contained the representative cycle nodes. Once again the position of the graph nodes in 2D were determined by the Fruchterman-Reingold algorithm from the igraph package. In order to color the PLRG nodes, the RDM is projected into 2D using the cmdscale MDS function from the R package stats (R Core Team, 2021), and the color of each node is determined by the location of its data point in MDS space according to a horizontal color scale [pink (left) to green (right)] and a vertical color scale [blue (downwards) to orange (upwards)]. For example, the top-right most coordinates would be an average (in RGB space) of the maximum vertical color, orange, and the maximum rightmost color, green, yielding brown. Outside of calculating the color of each node, the full visualization process of a PLRG is performed by TDApplied.

## 3 Results

### 3.1 Human and monkey IT cortex data and GoogLeNet

Using our approach, RTA revealed a significant difference in the topological strucutre of the RDMs between humans and monkeys—*p* < 0.001 in both *H*_0_ clusters and *H*_1_ loops. In order to capture what different loop structures may have existed in the two spaces, we identified their most persistent loop features, i.e. the loop with greatest difference between their death and birth values—this loop “lives” longest compared to all other loops. We will refer to these two most persistent loops as the “human loop” and “monkey loop”. We localized each loop by examining their respective representative cycle, i.e. a subset of the 92 points on each loop. Representative cycles are a useful tool for exploring topological features because those features may occupy distinct regions of the data space. For example, imagine a dataset with a loop and a cluster (not touching each other)—all the data points would be used to calculate the data's persistence diagram but only a subset of the points would lie on the loop. On the other hand, the points that do not lie on the loop, even if they are not part of any other interesting topological structure, can still have immense value in non-topological analyses (for instance in RSA analyses).

**Figure 6:**
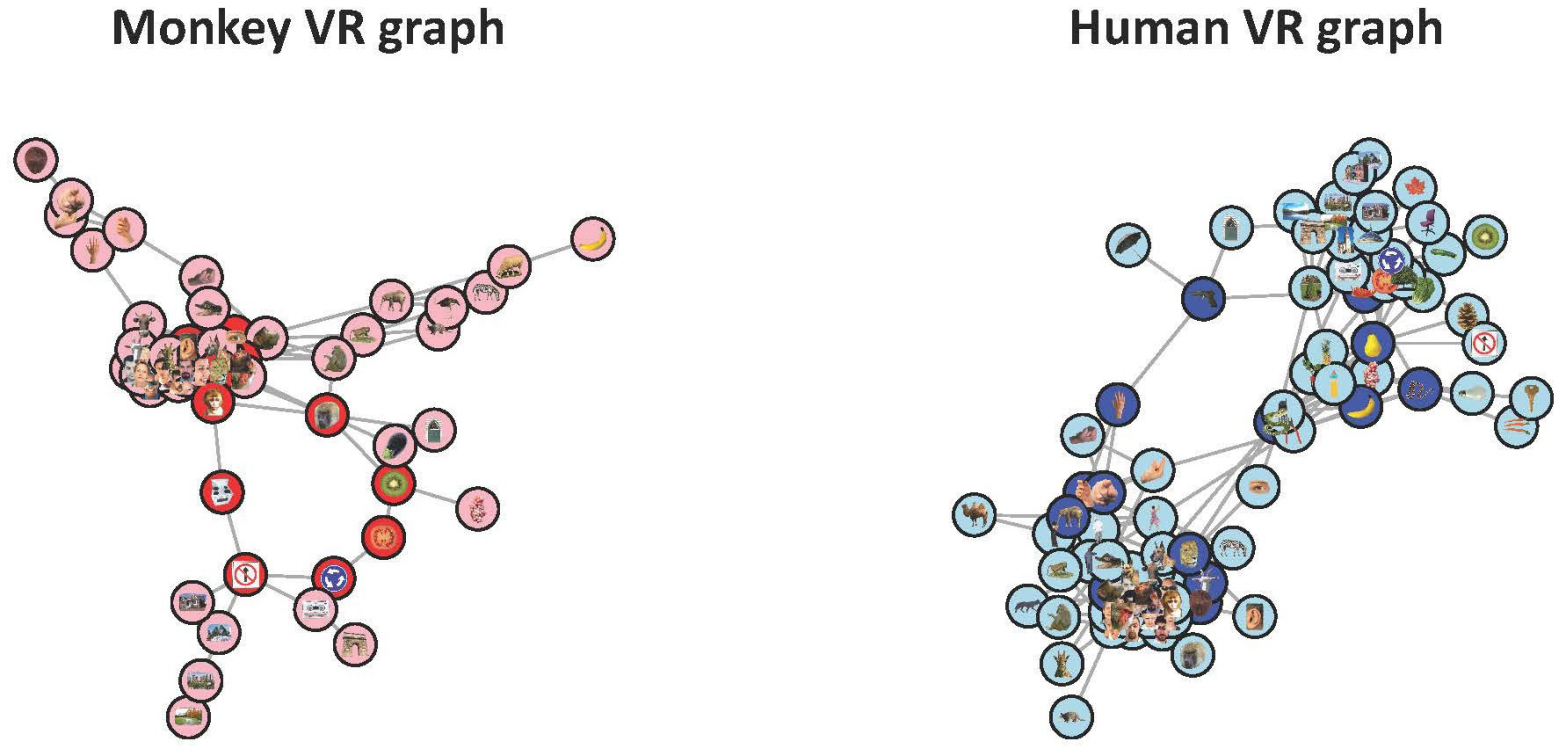
The PLRGs of the monkey RDM (**left**) and the human RDM (**right**) at the scales of their respective loop births. The monkey visualization shows a central cluster of animal and monkey faces, from which the loop and two flares (an animal body flair, right, and a hand flair, top left) stem from. From the loop there is also one flair which corresponds to scenery. Only 54 of the 92 stimuli were plotted as these vertices made up the connected component of the VR graph which contained the loop (each of the other 38 stimuli either had no connections to other stimuli or formed small, topologically uninteresting clusters). The human visualization contained 81 of the 92 stimuli, and appears to be two dominant clusters with two paths of sparse connections forming the loop. The clusters are animate objects (left) and inanimate objects (right).

To get a sense of the topological structure of the monkey and human RDMs at those linkage scales we plotted both space's PLRG in Figure 6.

Striking differences occur between the two representational spaces in this view—the monkey VR graph highlights substantially more clustered representations that we can easily label, such as animals, hands, faces, objects, etc., while the human representational spaces appear to be organized into two clusters symmetrically around a loop. That in both cases the representations appear to be lobes organized around a central confluence is intriguing, and may merit greater investigation. It is worth noting that RSA suggests that the monkey and human representations are highly comparable (Kriegeskorte et al., 2008b), finding a gross clustering into animate and inanimate objects in both human and monkey spaces, whereas RTA reveals the ways in which they are actually different.

**Figure 7:**
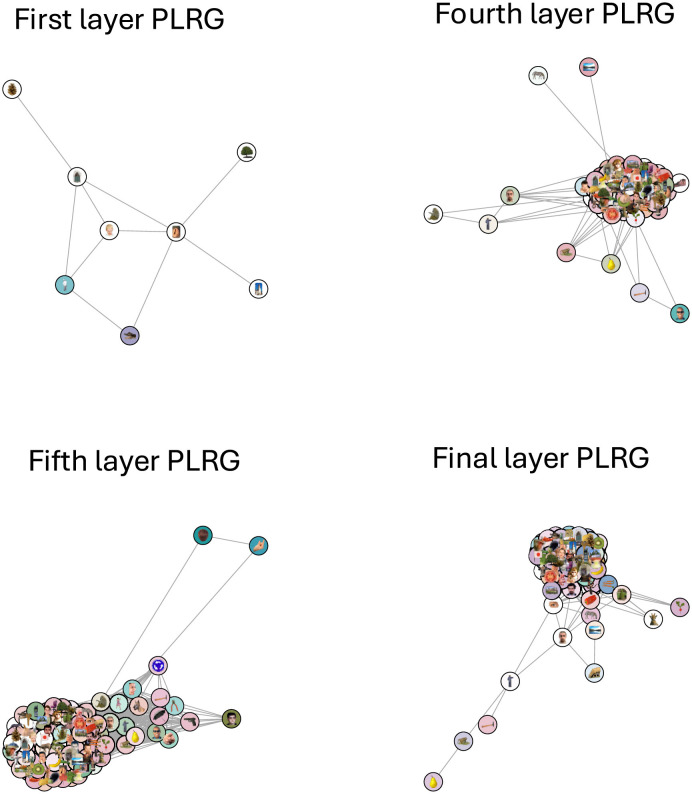
The four GoogLeNet PLRGs, in layers 1, 4, 5, and the final layer, each plotted at the birth radius of their largest loop. The first layer PLRG was mostly isolated stimuli (which were not plotted) and contained only a small loop, showing that continuous measures of object category likely do not exist at this early stage of processing. The three other PLRGs show a significantly more clustered representation of the images, with no clear pattern of the RSA-derived colors. Topologically, the learned structure of the representational space is resolved no later than the fourth layer.

Next we compared the neural network model GoogLeNet (Szegedy et al., 2015) against the human IT cortex data to see if the two systems were carrying out similar calculations. We fed the image stimuli through GoogLeNet and computed correlation distance RDMs from activity patterns in fully connected layers 1 through 6 (the final layer). None of the GoogLeNet RDMs had a significant correlation with the human RDM, and our model adjudication procedure found significant differences between each layer's topological structure and the human data's structure, both in terms of clusters or loops. In other words, both RTA and RSA agreed that the representational spaces were different. Out of the six new RDMs, only four of them had any loops, and in Figure 7 we plotted the PLRG of each of those four RDMs at the birth scale of their most persistent loop. These PLRGs show a very different structure to the human PLRG—the first layer space is largely sparse and disconnected, whereas later layers are one large cluster with some minor branching.

### 3.2 Naturalistic movie viewing data

**Figure 8:**
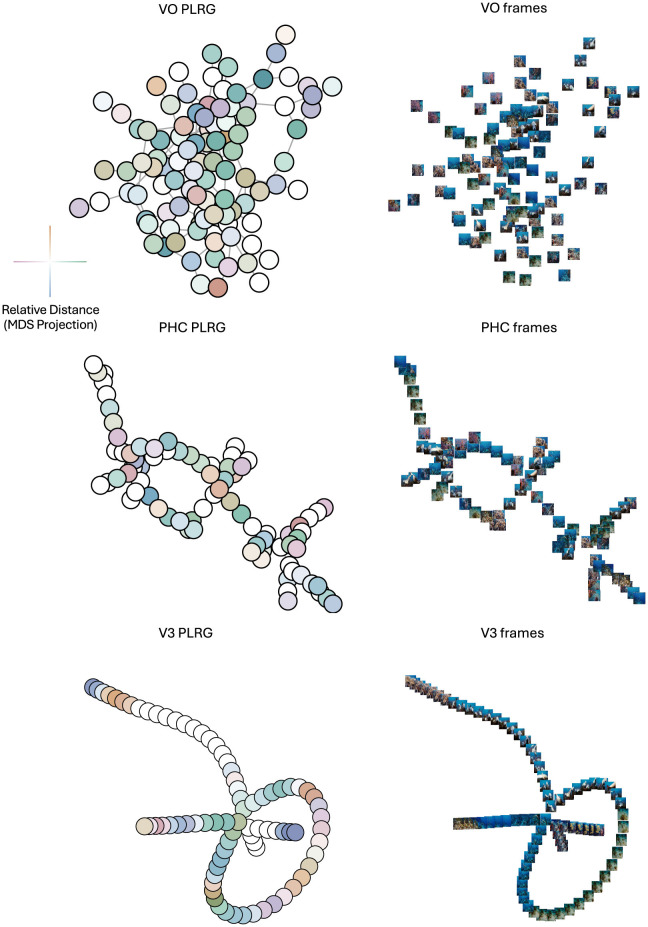
Topologies of mean representational spaces in VO (**top row**), PHC (**middle row**) and V3 (**bottom row**) areas. **Left column** is the PLRG laid out using a graph-layout algorithm, **right column** are the frames corresponding to each graph node, plotted at its node's 2D coordinate in the graph. The color-coding scheme for PLRG nodes, based on MDS coordinates, is displayed to the left of the VO PLRG—the x coordinate determines a horizontal color which is green for positive x-values and purple for negative x-values, and a vertical color which is orange for positive y-values and blue for negative y-values, and two nodes which have similar colors are TR's with correlated activity patterns, i.e. are nearby in MDS space. PHC and V3 have clearly-defined topologies in their PLRGs, whereas VO has mainly one densely-connected cluster. As well, the lack of color-clustering and smooth color gradients in the VO and PHC PLRG's indicate that MDS, i.e. RSA, did not capture the graph structure well. V3 on the other hand did exhibit color clustering and gradients, suggesting that there was a stronger relationship between topology and geometry at the loop birth scale. Moreover, the clustering and gradients suggest that some folding of the graph may be appropriate, where nodes which are far apart on the graph with similar colors may actually be proximal in terms of the geometry of data space. The frame visualization of V3 also appeared to most smoothly vary by color and scene type compared to PHC and VO.

RTA's model adjudication method found a statistically significant difference between the loop structures in each pair of the three representational spaces (at a Bonferroni-corrected level of α = 0.05/3). In order to determine what structures these differences may have be capturing, we plotted the VR graphs of the three mean RDMs, at the scale of their respective loop birth values, subsetted to contain only the data points which were in the components of their respective loop representative cycles. Since each graph represents data from one movie across subjects, each graph node represents a TR in its graph's movie, so each node is plotted with the movie frame five seconds prior to the TR (accounting for the hemodynamic lag). To determine if and where RSA provided a complementary view of these graphs, we projected the three RDMs (subsetted for the TRs in their respective VR graphs) into 2D space using MDS and colored each node in the VR graph by its location in MDS space. We call this novel visualization a *proximity-labeled rips graph* (PLRG for short). Finally, in order to link the PLRG's back to the raw data we also plotted the movie frame associated with each graph node at the node's location (in the graph space, not in the MDS space). The results can be seen in Figure 8.

The differences in the topological structure between the three regions can be readily appreciated, and these structures were not accounted for by MDS of the RDMs. This illustrates the importance of topological analysis of representational space for inference on similarity.

## 4 Discussion

We demonstrated the potential of topological analysis in identifying representational structures in stimulus-driven fMRI patterns, and showed how this knowledge of the representational geometry can be complementary to standard RSA. Importantly, sensitivity to topological features allows one to find non-linear dimensions (i.e. non-linear variables that capture meaningful variance in the structure of the data) in representational spaces, such as the loop we reported for the monkey IT data. This approach goes beyond classic inferential statistics and allows us to have insight into the nature of the mechanisms underlying neural representations.

We first examined two RDMs, one averaged from four human's IT cortices and one averaged from two monkey's IT cortices. Unlike in Kriegeskorte et al. (2008b) we found that the representational spaces were different, determined by RTA's model adjudication procedure, and we were able to visualize how the most persistent loops in the monkey and human representational spaces did not appear to encode the same information—the monkey loop likely encoded a continuous spectrum of change in object category, whereas the human loop was more likely the distal connections between animate and inanimate clusters. Two possible explanations of the differences between the two loops could be that (1) human IT cortex efficiently resolves object category into natural and animate clusters, whereas this distinction is more blurred (i.e. continuous) in monkey IT cortex, or (2) the representational spaces are distinct simply because humans and monkeys can have very different semantic encodings of the same image. The first explanation seems unlikely—the monkey VR graph also had a clear distinction between animate and inanimate objects. The second explanation seems more likely, for example a giraffe and a monkey may have similar representations in humans because they are both animals found in Africa, and in monkeys because they are both non-dangerous creatures—in other words, there is not a one-to-one semantic correspondence. This result is perhaps the best exemplar of the major criticism of RSA described earlier—the two species may be performing very different calculations, and this difference was only detectable using RTA.

The RSA and RTA comparisons of the GoogLeNet RDMs and the human RDM all agreed that the geometry and topology of the spaces were different, and these differences were clearly visible in the PLRGs—the model's first layer did not have any high-level object patterns resolved, whereas later layers were largely one dense cluster with some flairs, and the human data was better captured with two clusters connected by sparse connections along two paths. GoogLeNet was therefore likely capturing overly-complex image features compared to the biological data.

We carried out the same analytic approach to a naturalistic movie viewing dataset (Zhang and Farivar, 2020). We analyzed group-mean topological structures in VO, PHC and V3 areas. All three pairwise regional comparisons found significantly different loop signatures, and our novel proximity-labeled rips graphs of the spaces, at the scale of their most persistent (significant) loop's birth, were visually very different between the three regions and could not be accounted for by geometry alone. For example, the VO and PHC PLRGs did not have similar colorings of nearby nodes, and the V3 PLRG had clusters of similarly-colored nodes which existed far apart in the graph. By plotting the frames corresponding to 5 seconds prior to each TR over each TR's graph node, we can see that V3 is likely representing low-level movie features—like object position/movement or scene color, as demonstrated by the many neighboring frames which seem to only differ in the position of objects in the frame or the scene color. On the other hand, there is not a clear division of scene (object) category in the VO PLRG, nor is there a clear relationship between graph structure and object movement/position in the PHC PLRG, but the PHC does exhibit a clear structure (as opposed to VO). In this example RTA was also able to capture aspects of representational spaces which RSA could not.

While RSA provides a “hub” for researchers to integrate data from different modalities, species, etc., it may be limited by the requirement of fixed-size matrices to encode representational geometries and correlations. Because persistent homology

- is invariant under reordering its input data,
- can compare outputs regardless of the number of points it used as input, and
- its output converges as the number of data points grows,

representational topology analysis may be well-suited to compare RDMs across RSA studies which do not have the same stimulus set or set size, building on ideas first proposed in Laakso (2000). Concrete evidence for this use-case of RTA is hidden in our naturalistic movie analysis—free movie viewing does not follow the traditional task-based experimental design of RSA studies, which is necessary to compute stimulus-stimulus representational dissimilarities, but the flexibility of RTA allowed us to consider each time point as a “stimulus”, the spatial pattern at a time point its “representation”, and carry out principled comparisons of the topological structures which arose across subjects and movies. This type of RSA-like application, “time continuous RSA”, was discussed as a potentially interesting use-case of RSA in Kriegeskorte et al. (2008a). This means that fMRI datasets (as well as data from other functional neuroimaging modalities) can be compared regardless of their experimental design or duration, which would be particularly interesting for resting-state datasets. Resting state data is characterized by co-fluctuations between distal but functionally-related regions (Biswal et al., 1995; Cordes et al., 2000; De Luca et al., 2006), which implies the existence of periodic spatio-temporal signals that could be detected with persistent homology—spherical representational topologies have already been identified in resting-state (and naturalistic image viewing) electrophysiological data from V1 in monkeys (Singh et al., 2008) and this topology could be explained by the interactions between the (periodic) orientation and spatial frequency feature maps. To our knowledge RTA is the first framework that allows comparisons between scans of different duration and study design without temporally collapsing data.

Despite the unique capabilities of RTA, it does have several limitations. Firstly, it is more complicated than regular RSA—there are more computational tools which are needed to carry out a topological analysis. Secondly, RTA is more computationally demanding—persistent homology can be computed quickly with small RDMs (up to around 100 stimuli) in low dimensions, but computing higher-dimensional homology with large RDMs will likely be slower. Similarly, the analysis procedures for persistence diagrams can take time if the persistence diagrams contain many points (although this can be remedied by using the bootstrap procedure to only select significant topological features) or if there are a large number of persistence diagrams (as in a fMRI searchlight analyses). Thirdly, since persistence diagrams converge with larger sample size (Chazal et al., 2014), RTA will be better able to reliably capture topological features with more data points [RSA also performs better with more data (Kriegeskorte, 2009)]. Our informal assessment would suggest at least twenty data points as the low-end threshold.

Representational topology analysis directly addresses the topology of representational space—an aspect that RSA (as a linear geometric method) cannot. This understanding of representational geometry is useful in that it can reveal non-linear dimensionality of the representation space which has direct implications for the nature of the input patterns and, by extension, the mechanisms that give rise to those input patterns. In this manner, understanding the topology of representational space provides for novel insights unafforded by existing methods.

## Data Availability

The original contributions presented in the study are included in the article/supplementary material, further inquiries can be directed to the corresponding author.
